# Molecular and Cellular Modelling of Salivary Gland Tumors Open New Landscapes in Diagnosis and Treatment

**DOI:** 10.3390/cancers12113107

**Published:** 2020-10-24

**Authors:** Cristina Porcheri, Christian T. Meisel, Thimios A. Mitsiadis

**Affiliations:** Orofacial Development and Regeneration, Institute of Oral Biology, University of Zurich, Plattenstrasse 11, 8032 Zurich, Switzerland; christian.meisel@zzm.uzh.ch (C.T.M.); thimios.mitsiadis@zzm.uzh.ch (T.A.M.)

**Keywords:** salivary glands, head and neck cancer, epithelial tumors, pleomorphic adenoma, mucoepidermoid carcinoma, adenocarcinoma, transgenic animal models, organoids, organ-on-chip, molecular therapy, immunotherapy

## Abstract

**Simple Summary:**

This review elaborates the current knowledge on salivary gland tumors, with a specific focus on classical histological classification, cellular mechanisms and molecular pattern at the origin of the most common glandular malignancies. We dive into novel approaches for modeling, diagnosis and therapy, giving an overview of the biomedical advances for the study of salivary cancers. Thereby this review helps to understand the complexity of these malignancies and paves the way for novel and efficient treatments.

**Abstract:**

Salivary gland tumors are neoplasms affecting the major and minor salivary glands of the oral cavity. Their complex pathological appearance and overlapping morphological features between subtypes, pose major challenges in the identification, classification, and staging of the tumor. Recently developed techniques of three-dimensional culture and organotypic modelling provide useful platforms for the clinical and biological characterization of these malignancies. Additionally, new advances in genetic and molecular screenings allow precise diagnosis and monitoring of tumor progression. Finally, novel therapeutic tools with increased efficiency and accuracy are emerging. In this review, we summarize the most common salivary gland neoplasms and provide an overview of the state-of-the-art tools to model, diagnose, and treat salivary gland tumors.

## 1. Introduction

The salivary glands are organs belonging to the orofacial complex whose epithelial structures are specialized in the production and secretion of saliva [1]. Aberrations of salivary glands upon irradiation exposure, autoimmune diseases, viral infections, and cancer, result in a limited or complete abrogation of saliva production, which greatly impacts speech, taste, and feeding function. Cancers of the salivary glands represent 6% of all head and neck cancers and greatly vary in origin, subtype, and behavior (Table S1) [2,3]. Most of these tumors are benign and might evolve into a malignant form after several years from their initial diagnosis. Previous exposure to irradiation is one of the most common causes of the salivary gland cancers, although viral infection and prolonged chemical usage correlate with a high incidence of glandular tumors [4,5,6,7].

The parotid (one of the major salivary glands) is most frequently affected by cancer (85% of the cases), followed by the submandibular glands and the minor salivary glands of the palate [8,9]. The sublingual gland is seldom affected by cancer (1% of the cases). The tumor forms a firm and nodular mass, which at the initial stages is normally painless [10]. However, the cancer can spread to the facial nerve, causing numbness, paresthesia, loss of motor function, and pain [11]. The 5-year survival rate of patients suffering from salivary gland tumors is around 71%, with great variability depending on the type of tumor, site of appearance, aggressiveness, and stage [9]. Upon malignant transformation glandular cells can acquire a metastatic behavior, with the cervical lymph nodes, lungs, liver, and bones being the most targeted organs.

Accurate identification of the various subtypes of salivary gland tumors can be challenging due to their shared features and strong histological overlap [12,13]. Recently, novel tools for precise diagnosis and personalized medicine have emerged and can be instrumental for the development of new and more efficient treatments.

## 2. Benign Tumors

### 2.1. Pleomorphic Adenoma

**Figure 1:**
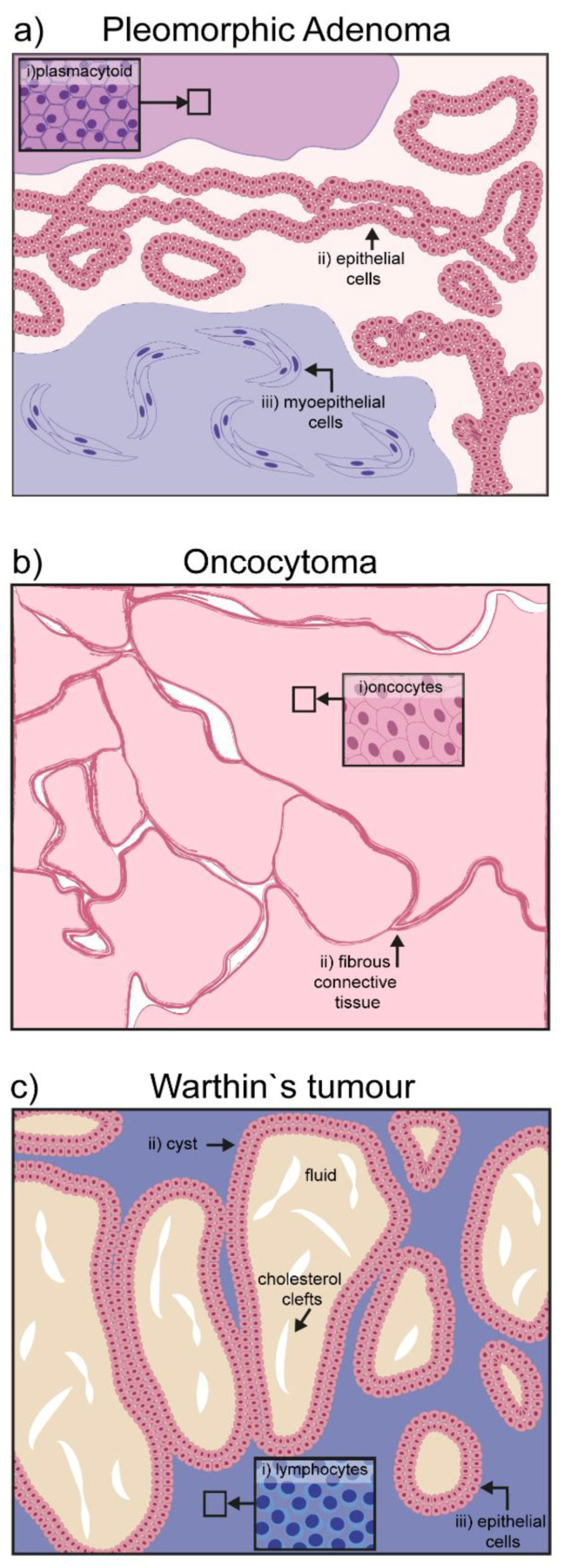
Schematic representation of hallmarks in benign salivary gland tumors: (**a**) Pleomorphic adenoma is characterized by three different cell types: (i) Plasmacytoid, (ii) epithelial cells, and (iii) myoepithelial cells; (**b**) oncocytoma is composed of a homogeneous mass of oncocytes bordered by fibrous connective tissue; (**c**) the papillary cystadenoma lymphomatosum or Warthin’s tumor, is characterized by its cystic components filled with amorphic fluid and occasional presence of cholesterol clefts; the major cellular components are lymphocytic infiltrates surrounding the cyst.

Pleomorphic adenoma (PA) is the most common salivary gland neoplasm worldwide, accounting for 70–80% of abnormal growths [14,15]. It mainly occurs in the superficial lobe of the parotid gland, but can also affect the submandibular and minor salivary glands. It forms a painless bulging mass of epithelial and myoepithelial tissues with minor stromal and mesenchymal components [16]. Epithelial cell clusters form characteristic ductal or sheet structures, while myoepithelial cells become a continuous part of the stroma, which in turn can acquire mucoid or chondroid features. Rarely, myoepithelial cells differentiate into plasmacytoid cells, a peculiar cytological characteristic of mixed salivary tumors (Figure 1a) [17].

Pleomorphic adenoma can transform into carcinoma (CXPA-Carcinoma ex pleomorphic adenoma) with an aggressive progression in 6% of cases [12]. This transition is accompanied by a switch in metabolic need of the growing mass, which abandons its dependency to glucose to adopt a long-lasting source of energy, such as fatty acids [18]. Benign tumors can be resected, but often reappear with a multifocal pattern. Over the years, the rate of recurrence decreased dramatically, mainly due to the technical advances in surgical procedures, which now tends to maintain the tumor capsule intact [19].

### 2.2. Oncocytoma

Oncocytoma is a rare group of benign tumors of the salivary glands mainly found in elderly patients (over 60 years old). Although the parotid is the gland with the highest incidence (84% of the cases), some cases have been reported involving the submandibular or sublingual glands [20] The encapsulated painless mass grows slowly and it is composed quite exclusively of oncocytes. Oncocytes are polyhedral epithelial cells, double the size of normal acinar cells, characterized by a condensed round nucleus and a mitochondria-rich cytoplasm that is positive for phosphotungstic acid–hematoxylin (PTAH) staining (Figure 1b) [21]. Secretory epithelia exposed to metabolic stress might evolve into oncocytes to compensate new energetic needs [22]. The cause of the transformation is not completely clear. Aging itself may be at the basis of the oncocyte transformation, as mitochondria enzymes become ineffective over time, and an overcompensation is put in place by increasing their total number [22]. Viral infections such as Epstein–Barr (EBV), HIV, herpesvirus, and papillomavirus can also be associated with the epithelial transformation into oncocytoma [23]. Surgical intervention is the most common therapeutic approach, which results in total or partial parotidectomy.

### 2.3. Papillary Cystadenoma Lymphomatosum (Warthin’s Tumor)

The papillary cystadenoma lymphomatosum is a benign tumor affecting almost exclusively the parotid gland. Sometimes painful, it is mostly asymptomatic and mainly produces side effects due to the swelling process, such as earache, deafness, tinnitus, and facial alterations. It is usually an encapsulated cyst, containing amorphic material, with some cholesterol clefts [24,25]. The altered salivary gland tissue presents stromal fibrosis and an oncocytic mass with prominent lymphocytic infiltrates (Figure 1c) [24]. The origin of the tumor is still debated, although hyperplasia is most probably derived from increased proliferation of ductal cells in the salivary gland [26,27]. On the other hand, it is unclear if the lymphoid component derives from altered lymph nodes, or occurs secondarily due to a lesion in the epithelium, or a combination of both [24,28]. Aside from B- and T-cells, the infiltrate also contains macrophages and mast cells, suggesting the local presence of an antigen triggering immune cell recruitment. EBV infection has been detected in the tumorigenic tissue, and might represent the antigenic stimulus for immune cell recruitment [28].

Surgical excision of the mass is an efficient procedure as the intact capsule can be preserved, and only 1–15% of resections are followed by recurrence of the tumor [29].

## 3. Malignant Tumors

### 3.1. Mucoepidermoid Carcinoma (MEC)

**Figure 2:**
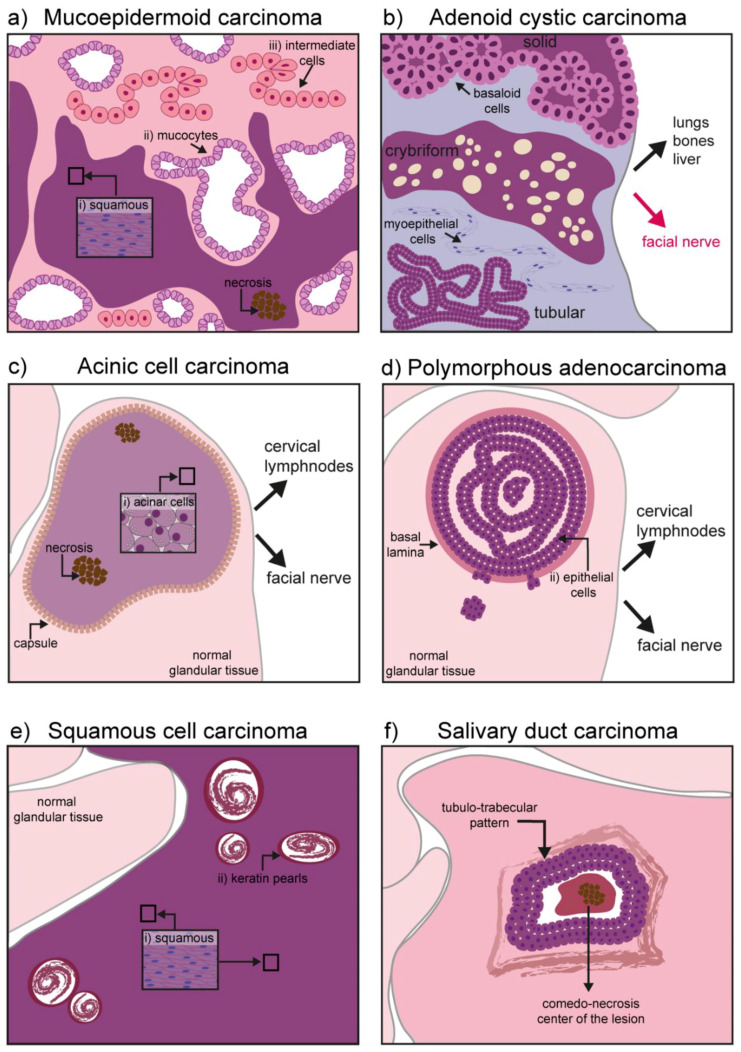
Schematic representation of hallmarks in malignant salivary gland tumors: (**a**) Mucoepidermoid carcinoma might contain three different types of cells depending on its grade: (i) Squamous (epidermoid) cells, (ii) mucocytes, and (iii) intermediate cells. High grade tumors also show necrosis and invasiveness to neighboring tissues (neural and bones); (**b**) adenoid cystic carcinoma has three different forms: Solid, cribriform, and tubular. Basaloid, tubular, and myoepithelial cells are commonly found. It has an important neurotropism, and cancerous cells can form distal metastases in the lung, bones, and liver; (**c**) the acinic cell carcinoma is characterized by a homogeneous mass of differentiated acinar cells rich in granuli. In primary tumors, the mass is encapsulated while the capsule might disappear in recurrent tumors. Although seldom, upon recurrence the tumor might contain areas of necrosis and it can acquire invasiveness to local lymph nodes and the facial nerve; (**d**) the polymorphous adenocarcinoma consists of a confined mass, although not encapsulated. Epithelial cells form the core of the tumor mass. High-grade tumors can digest the basal lamina and invade the gland parenchyma. Invasiveness to cervical lymph nodes and the facial nerve has also been reported; (**e**) squamous cell carcinoma is mainly formed by squamous cells and contains keratin pearls derived from epithelial differentiation and accumulation of keratin; (**f**) salivary duct carcinoma is characterized by a comedo-necrotic core surrounded by ductal cells. Subforms (not depicted) include mucin-rich, papillary, micropapillary, sarcomatoid, and oncocytic.

The mucoepidermoid carcinoma is a hyperproliferation of excretory cells affecting bronchi, thyroid gland, eustachian tubes, lacrimal, and salivary glands [30]. Concerning the salivary glands, this malignant tumor represents the 30–34% of all salivary gland cancers, having a predilection for the parotid glands [10,31]. Three types of cells can be found in this tumor: Mucocytes, intermediate cells, and squamous (epidermoid) cells. Mucocytes have a foamy, lightly colored cytoplasm, with a small acentric nucleus and form clusters giving rise to duct-like structures. Epidermoid cells remain sparsely distributed and can be recognized by their intense eosin staining. Intermediate cells are common in these tumors and often retain the appearance of lightly eosinophilic basal cells [32]. Depending on the grade of the tumor, the proportion and cellular composition of the mass varies: In low-grade tumors there is a predominance of mucous cells, while epidermoid and undifferentiated cells populate high-grade tumors. High-grade tumors are also characterized by necrotic lesions and high invasiveness, particularly to the neural tissue and the neighboring bone [33] (Figure 2a).

To date, the etiology of MEC is unknown. Papilloma virus (HPV) infection has been reported in neoplastic regions but its role in sustaining the tissue lesion is still under debate [34,35]. Similarly, the cytomegalovirus (CMV) has a specific tropism for salivary glands and can produce chronic infections in the ductal epithelium [36,37].

Early diagnosis is a central element to hinder MEC growth and prevent metastatic spread. Fusion proteins derived from chromosomal rearrangement have been found in 64% of MEC cases and constitute a major diagnostic and prognostic factor [38] (see also 4.1 and Table S1).

### 3.2. Adenoid Cystic Carcinoma

Adenoid cystic carcinoma (AdCC) is a glandular form of adenocarcinoma encountered in the breast, uterus, skin, and most commonly in major and minor salivary glands. It accounts for 7.5–10% of all salivary gland neoplasms [39,40]. It is characterized by a marked neurotropism, often invading the facial nerve causing pain and paralysis [11]. Contrary to most carcinomas it rarely metastasizes to closely located lymph nodes, but rather to the lungs, liver, or bones. Due to the strong interconnection with the facial nerve, surgical excision is challenging and often accompanied by radiotherapy to compensate for incomplete resection. Relapsed tumors are often incurable and their molecular signature largely unknown. Recently, a comparative analysis of genomic alteration was performed between primary and recurrent/metastatic AdCC. The specifically mutated genes included the Notch pathway, chromatin remodeling genes, DNA damage repair, and tumor suppression genes [41].

This cancer can affect myoepithelial cells as well as ductal cells. Histological patterns define the three most common forms of the tumor: Tubular form, cribriform, and solid form [42,43]. The tubular form has the best prognosis and it is formed by elongated structures of epithelium lining a central lumina. The cribriform is characterized by the presence of cells grouped around a microcyst. The solid form is the most aggressive one, with a reduced level of myoepithelial differentiation, presence of basaloid and ductal epithelial cells, increased cell proliferation, and poor prognosis (Figure 2b) [43].

### 3.3. Acinic Cell Carcinoma

Acinic cell carcinoma (AciCC) is the third most common epithelial malignancy of the salivary glands in adults and the second most common in children [44].

It progresses slowly (low-grade behavior), but can metastasize to local and distant sites upon recurrence (i.e., cervical lymph nodes and lungs). Primary tumors are soft and encapsulated, while their appearance changes significantly upon recurrence, with reappearing tumors characterized by a degraded capsule (Figure 2c) [44]. A subset of AciCCs have recently been reclassified as secretory carcinomas, morphologically similar to AciCC, but characterized by the ETV6-NTRK63 gene fusion [44,45]. Although irradiation is sometimes used as adjuvant therapy to avoid recurrence, exposure to radioactive isotopes is of controversial usage and requires strong consideration of risks versus benefits. In fact, exposure to radiation has been identified as one of the major risk factors in AciCC development [44,46]. Historical cases of exposure to radiation following atomic bombing during world war II (WWII) are linked to development of AciCC. More recently, the usage of the isotope Iodine 131 for the treatment of head and neck cancer has been shown to specifically accumulate in the salivary glands and was linked to the development of the AciCC tumors [32].

### 3.4. Polymorphous Adenocarcinoma

Polymorphous adenocarcinoma (PAC) is an epithelial tumor encountered in several regions of the oral cavity, and particularly in the minor salivary glands [47,48]. It has an indolent course and it is characterized by cytological uniformity. The mass is not encapsulated, but usually remains confined in a region for years. It grows within the epithelial structure and frequently disrupts the basal lamina to invade the surrounding parenchyma. Although the risk for distal metastases is relatively low, cancer cells can invade the cervical lymph nodes as well as the facial nerve, with a typical perivascular disposition (Figure 2d) [48,49,50,51].

### 3.5. Squamous Cell Carcinoma

Squamous cell carcinoma (SCC) is one of the most common cancers of the oral cavity, but the salivary glands are rarely affected by this malignancy. The exact frequency of primary tumors in salivary glands remains unknown, as cancer cells from skin squamous cell carcinoma might use glandular tissue as a secondary site for metastasis. The tumor is of epithelial origin arising mainly in the parotid gland and it is seldom encountered in the submandibular gland tissue [52,53]. Ductal or cuboidal epithelium undergoes a metaplasia transformation and progress into a carcinoma infiltrating the gland parenchyma (Figure 2e).

Previous exposure to irradiation is accounted to be the major risk factor for the development of glandular SCC, followed by viral infections. Infection of HPV of the oral cavity might extend preferentially to the parotid via the Stensen duct, where cellular transformation has been linked to the alteration of the PTEN/Pi3K pathway [54].

The most efficient therapeutic approach is the resection of the tumor mass; in case SCC is found in the submandibular gland, also the cervical lymph nodes are resected to diminish the probability of metastases.

### 3.6. Salivary Duct Carcinoma

Salivary duct carcinoma (SDC) is an aggressive adenocarcinoma accounting for up to 2% of salivary epithelial malignancies. It mainly affects the parotid, although cases where the submandibular glands and minor salivary glands were affected have been reported [55]. SDCs often arise de novo but can also constitute the malignant part of a carcinoma ex-pleomorphic adenoma. It has a high local recurrence rate and can form distal metastases [56].

Although the morphological appearance can be variable (ranging from mucin-rich, papillary, micropapillary, sarcomatoid, and oncocytic), SDC cancer cells are usually characterized by ductal differentiation, with a classic comedo-carcinoma pattern and a periductal rim (Figure 2f) [57].

Morphologically, it shares strong similarities with the high-grade form of breast ductal carcinoma, and similarly to the breast malignancy, most cases have a high level of HER2 and cross cystic disease fluid protein-15 (GCDFP-15) [58,59]. An important difference between SDC and breast ductal carcinoma is the expression of the hormone receptor profile: Breast cancer is often characterized by a high expression of progesterone receptor and the α-isoform of the estrogen receptor, both almost absent in SDCs. On the other hand, SDC often shows a high expression of the androgen receptor (AR) and the β-isoform of the estrogen receptor [59,60]. Some similarities with the prostatic carcinoma also exist, as both frequently express TGFα, EGFR, and peroxisome proliferator-activated receptor gamma (PPARγ) [59,60].

## 4. Cellular and Molecular Mechanisms at the Origin of Salivary Gland Tumors

### 4.1. Genetic and Molecular Deregulation in Salivary Gland Tumors

Aberrant regulation of growth factors, morphogens, and cell-to-cell communication signals has been identified in cancer of the head and neck region. Tumorigenic conditions largely recapitulate events occurring during development, and molecular pathways that are essential during embryonic life can be reactivated to increase proliferation, self-renewal, and survival in cancerogenic conditions.

The WNT/β-catenin pathway, for instance, is involved in cell fate determination and stem-cell maintenance. Importantly, β-catenin is delocalized in pre-cancerogenic lesions and oral squamous carcinoma, and might be involved in the regulation of adherens junction anchoring hotspots [61,62]. The salivary gland form of SCC is characterized by an epithelial hyperplasia, where the amplification of the undifferentiated progenitors correlates with a high level of WNT/β-catenin and a low level of BMP signaling. Specifically, β-catenin activation triggers epigenetic modifications of stem-cell genes promoter, regulating their transcription [63]. The WNT/β-catenin pathway has been involved in other salivary gland tumor etiopathogenesis, such as AdCC [64]. Epithelial cells express high levels of E-cadherin, which functions as a crosslink between cells. E-cadherin directly regulates the activity of β-catenin by keeping it localized to the membrane and preventing its translocation to the nucleus. Thus, reduction in E-cadherin levels may result in a dysregulation of the WNT/β-catenin pathway activity, ultimately inducing high motility and increased proliferation [64,65].

The activation of oncogenic pathways generally results in the recruitment of specific transcription factors, such as the morphogenesis regulator AP2γ. Analogously to other ductal tumors (e.g., mammary gland tumors), AP2γ is overexpressed in AdCC samples [66]. It directly regulates the expression of the receptor for the stem cell factor kit, another tumorigenic marker that is highly expressed in AdCC [67].

Similarly to WNT, the NOTCH pathway is a crucial element to balance regeneration and differentiation in both embryonic and adult tissues. Its central role in cancer spans from induction of fate transformation and activation of a pro-tumorigenic environment to dysregulation of the immune system. In head and neck squamous cell carcinoma, NOTCH plays a pivotal role by increasing cell proliferation and tumor growth [68,69,70,71].

Chromosomal translocations involving the NOTCH-pathway co-activator MALM are hallmarks of MEC [38]. The chromosomal translocation t (11;19) (q21;p13) producing the fusion protein MALM2/METC1, where METC1 is a coactivator of the cAMP response element-binding protein (CREB), has been detected in the early stages of the tumorigenic process [72,73,74,75]. This fusion product regulates the differential expression of downstream targets, such as HES1 and HES5, FLT1 and NR4A2, and its presence correlates with clinical-pathological indolence and an important increase in the survival rate (over 10 years compared to 1.6 years in the absence of the fusion product) [74,75]. Additionally, a lower risk of metastases and recurrence was associated with a positivity for MALM2/METC1 [74,76]. Molecularly, the fusion protein has been shown to regulate the activation of the EGF pathway via its ligand AREG [77,78,79,80]. Both fusion products and EGF expression are used for diagnostic purposes to predict the outcome of the disease. FISH analysis of MALM2 rearrangement has been used to identify MEC in histological samples that strongly resembled Warthin’s tumor [81,82]. Chromosomal rearrangements have also been described in pleomorphic adenoma, such as the reciprocal translocations t (3;8) (p21;q12) resulting in promoter swapping between the zinc finger PLAG1 and the β-catenin gene [83]. In adenoid cystic carcinoma, translocation of t (6;9) (q22–23;p23–24) induces activation of the transcription factor MYB [84], while secretory carcinomas are characterized by the t (12;15) (p13;q25) translocation resulting in the ETV6-NTRK3 fusion gene [13]. The ETV6 is a suppressor of transcription, while the (tropomyosin receptor kinase C-NTRK3) codes for the tyrosine kinase domain of the membrane receptor. Ultimately, the chimeric protein ETV6-NTRK3 promotes growth, survival, and proliferation [84,85,86].

Strong parallelisms exist between the chromosomal rearrangements at the basis of different glandular tumors. Particularly, gene fusion involving MYB, MYBL1, and NFIB have been described in salivary, lacrimal, and triple-negative mammary AdCC, although the clinical outcome of these tumors can greatly vary [87]. Chromosomal loss such as depletion of the regions 6q23–q27, 12q12–q14, and 1p32–p36 are also common in AdCC, with the latter occurring in 44% of cases and are linked to shorter survival rates [88]. These findings indicate that important tumor suppressor genes associated with the onset or early stages of AdCC are harbored in the 1p36 chromosome. p73 and Ve-cadherin (CDH5), both map in this region and are responsible for p53-regulation of apoptosis, proliferation, and senescence [89,90]. Screening analyses of patients’ samples also revealed an up-regulation of the transcription factor SOX4 together with members of the NOTCH and WNT pathways [66,91,92].

Recurrent rearrangements of t (4;9) (q13;q31) have been found in AciCC, resulting in a specific upregulation of the nuclear receptor subfamily 4 group A member 3 (NR4A3). Overexpression of NR4A3 triggers proliferation of glandular cells most probably regulating the downstream expression of CyclinD1 [93]. Additionally, the gene fusion HTN3-MSANTD3 (Histatin3 and Myb/SANT-like DNA-binding domain containing 3) was linked to 3% of the cases analyzed and associated with the development of a metastatic behavior [94,95]. Importantly, other genetic markers have been exclusive, such as the high expression of *p53* in breast cancer, which remains low in salivary AciCC and constitutes a valuable diagnostic signature [96,97].

Several genetic alterations have also been associated with the progression of SDC [98]. The HER2 oncogene mapped in the 17q chromosome locus encodes for a membrane receptor protein capable to heterodimerize with any of the HER-family proteins and activate the downstream tyrosine kinase cascade [99]. Genetic profiling identified the HER2 gene amplification in SDC patients with poor prognosis and high rate of cell proliferation [100]. Similarly, the mutated or overexpressed form of TP53, is associated with the more aggressive form of SDC characterized by early local recurrence and distant metastasis [101].

Molecular dysregulation can also be accounted for the establishment of a pro-tumorigenic environment. PAC development, for instance, has been linked to a change in metabolism, mainly due to the increased expression of markers for autophagy. This cellular mechanism allows survival in hypoxic conditions and nutrients depletion, and it is linked to the expression of the autophagosome proteins Beclin and LC3B. In parallel, pro-survival markers (such as BCL2 and Survivin) and growth factors such as FGF2 and PDGFα and PDGFβ are overexpressed, while senescence signals are low (e.g., expression of p21 and p16) [102,103].

Finally, deregulation of specific molecular pathways correlates with a distinct pathological outcome and can be used as diagnostic hallmarks. Certain Aquaporin (AQPs) subtypes are specifically expressed in MEC tumors. AQP1 was found in the tumor vasculature, but it was absent in the parenchyma, while AQP3 and AQP5 were found in mucous and intermediate cells [104]. AdCC secretes a high number of extracellular matrix proteins, such as Collagen IV, Laminin, and Keratin 17, and shows double positivity for p63 and p40 that are used for diagnostic purposes [105,106,107,108]. Importantly, the p63-positive/p40-negative phenotype is instead characteristic of polymorphous adenocarcinoma [109]. A comparative table including genetic and molecular alterations in the salivary gland tumors is presented (Table S1).

### 4.2. Cancer Stem Cells in Salivary Gland Tumors

**Figure 3:**
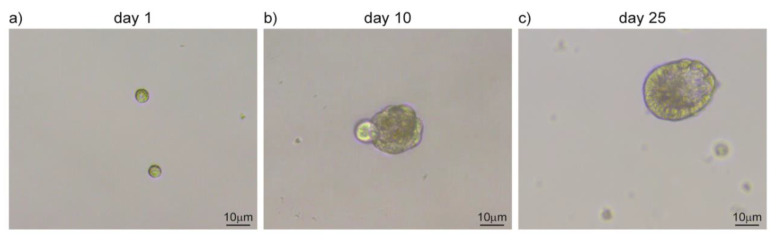
Salispheres culture from adult salivary glands. (**a**) Single-cell suspension upon submandibular gland dissociation. (**b**) Cells amplify in vitro and cluster together forming salispheres. (**c**) Salispheres grow larger and start to differentiate in duct-like structures. Original images from our lab.

The origin of salivary gland tumors vary greatly in between subtypes and both differentiated and precursor cells can participate in cancer initiation. Endogenous stem cells are present in the healthy salivary gland tissue and are characterized by a self-renewal ability and a privileged interaction with their supportive niche [110,111]. In aberrant conditions, adult stem cells can exploit these unique characteristics by establishing the undifferentiated core of the tumor. On the other hand, differentiated cells can be the target of transforming mutations that revert them in a stem cell-like subpopulation, with a similar outcome [112,113]. Independently from their origin, cancer stem cells are characterized by a slow cell cycle progression and persistance in a quiescent state. Due to their low proliferation rate, this subpopulation is only marginally targetted by chemotherapy, which instead acts mainly on the hyperproliferative portion of cancer cells [105,114]. Salivary gland tumors are resistant to chemotherapy and can recur upon resection. These characteristics support the hypothesis of cancer stem cells being at the origin of salivary gland tumors and might account for their peculiar resilience toward treatments [105,114,115]. Tumor relapses and the ability to form distal metastases are also common features of these types of tumors. Cancer stem cells might be able to reinitiate tumor growth in the primary site as well as upon migration to distal tissues. In support of this hypothesis, cells expressing undifferentiation markers (Kit, CD44, CD133, and ALDH) have been identified in MEC and AdCC [116,117,118,119]. Cells derived from salivary glands can be grown in vitro to form three-dimensional structures, such as salispheres, organoids, and tumoroids (Figure 3) [120,121]. Organoids retain the three-dimensional organization and the complexity of cell-to-cell and cell-to-microenvironment interactions, largely preserving cancer features [122]. They can be maintained in culture for several passages, allowing the amplification of the patient-derived cells and their usage for screening and manipulation [123]. In adequate conditions, stem cells forming salispheres can be differentiated in vitro to acquire the features that characterize their physiological maturation (Figure 3). Similarly, cancer-derived stem cells can be used to form organoid and model aberrant transformations that closely mimic tumor growth [124]. The MEC tissue from patients has also been used to produce orospheres that can regenerate themselves in vitro and form tumors when transplanted into immunocompetent mouse models [125]. The existence of a subpopulation of cells with stem cell-like features can therefore be used as a new tool for more efficient clinical approaches and novel diagnostic and modeling studies to recapitulate the disease in vitro [126].

## 5. Novel Approaches for Modelling, Diagnose, and Therapy

### 5.1. Traditional Approaches for Diagnosis and Therapy

Although slowly progressing, malignant salivary gland tumors can have a lethal outcome. Initial-stage tumors have a 5-year survival rate above 70%, while this percentage drops dramatically at more advanced stages (25%, 21%, and 23% for stages II–IV, respectively [9,13,127]. Surprisingly, the prognosis of patients remained unchanged in the last 30 years, thus reflecting the limited progress in the understanding of the origin and evolution of salivary gland cancers [31,127,128].

The most common treatment for salivary gland tumors is the surgical intervention. This corresponds to either the resection of the whole organ or the excision of a variable portion of the gland. Several side effects are associated with this approach. The complete resection of the tumor is a challenging procedure, even when the tumor is confined and encapsulated. This presents an important limitation as it allows the cancer to rebuild itself upon incomplete resection and often re-emerges in a more aggressive form. In some cases, the neurotropism of the tumor induces its growth in close proximity with the facial nerve, and surgical intervention is additionally complicated by the possibility of damaging the nerve, with devastating consequences [129]. Furthermore, resection of the primary tumor has limited impact in the presence of metastases.

Associated with the surgical intervention, radiotherapy is often used to prevent spreading of the potentially remaining cancer cells. However, the effects of irradiation on the tissue are largely unknown. Notably, salivary glands are particularly sensitive to irradiation compared to other organs, and display important effects on their physiology already at low doses and after a short time [130]. Radiotherapy for head and neck cancers is an important cause for salivary glands dysfunction, resulting in hyposalivation and xerostomia that can only be treated symptomatically with local administration of lubricants [131]. This classical therapeutic approach has developed into a more precise radiotherapy that uses protons rather than photons, which grants a lower exposure of the normal tissue and thus minimizes side effects [131]. Similarly, intensity-modulated radiotherapy (IMRT) is based on a more accurate delivery of radiation exposure, in the attempt of sparing the healthy tissue [131].

A more detailed knowledge of the cellular and molecular mechanisms governing tumor growth in salivary glands, therefore, is paramount to develop efficient tools for their containment and eradication. Based on the recent technological advances in cancer therapies, novel options are arising with promising results in the specific field of salivary gland tumors.

### 5.2. Perspectives in Biomedical Research and Diagnostics

#### 5.2.1. Organotypic Models

In the perspective of understanding the basic molecular and cellular events governing salivary gland tumors, novel in vitro models, organ-on-chip, and stem cell-based approaches represent the avant-garde of biomedical research.

Tumoroids largely recapitulate cell-to-cell and cell-to-ECM interactions of the tumor mass and allow studies on cancerogenic conditions. Additionally, they represent an accessible platform where the patient’s derived samples can be amplified and used for screening and pharmacological testing [123].

As our knowledge on salivary gland stem cells expands, more accurate models can be developed with the aid of biomimetic templates, where cells can be seeded to allow in vitro regeneration of fully functional organs [132]. Finally, a humoral and fluid-borne component that sustains the tumor microenvironment, can be studied with dynamic microchip plates where whole organs and salivary gland-derived cells can be maintained and manipulated according to experimental needs [133,134]. In the organ-on-chip approach, vascularization, innervation, and immunoreaction are part of the modelling system, and their influence is taken into consideration to more precisely mimic the complex interactions between tissues in the native organ [135]. These novel approaches do not only allow studying alterations in the physiology of salivary glands, but can also be used to observe the behavior of patient-derived cells. The accumulation of genetic mutations, proliferation, and self-renewal rate of patient-derived cells can be investigated in an accessible platform that allows screening and drug testing in a personalized therapeutic approach.

#### 5.2.2. In Vivo Models

One of the limitations in studying salivary gland tumors is the lack of appropriate models to recapitulate the main characteristics of the human disease. Mouse models are the gold standard for both basic biology and medical research in the development of novel therapies. Transgenic murine lines are now available to mimic basic aspects of salivary gland cancers. The oncogene KRAS can be overexpressed in a Tamoxifen-controlled manner under the control of the protease Elastase promoter, producing a phenotype that strongly resembles a salivary carcinoma [136]. Specific murine models for AciCC have also been developed based on the inhibition of Apc and Pten transcription under the control of the ductal MMTV [137]. Recently, a transgenic-driven knock-out for Pten and Smad4 under the control of the Keratin5 promoter, was shown to induce several types of salivary gland tumors (i.e., AdCC) [138]. Accurate in vivo models will allow us to study the cellular and molecular mechanisms of the disease from the early to the most advanced stages, such as invasiveness and metastasis formation. Additionally, upon systemic administration of drugs, side- and on-target-effects can be accurately monitored over time. Developing the most appropriate in vivo model, therefore, remains of high priority in the search of new treatments.

#### 5.2.3. Novel Diagnostic Tools

Diagnosis of the proper subtype of salivary gland tumors represents a major challenge to define the most appropriate and efficient therapeutic approach. Chromosomal rearrangements and gene fusions are particularly common in salivary gland tumors, and are important elements in the identification of specific cancer subtypes. At the side of traditional diagnostic tools, such as IHC and FISH detection, the next-generation sequencing (NGS) approach has been successfully applied in the recent years to extend and refine our knowledge on salivary gland tumors [139]. NGS approaches have been used for whole exome, transcriptome, or specific-target sequencing, to generate a precise barcoding of neoplastic samples. This massive acquisition of data allowed the identification of previously unknown fusion genes, which can be used as diagnostic tools as well as novel targets for therapeutic development. With this system, equivocal FISH results derived by complex and heterogeneous samples could be further investigated. For instance, the most common salivary gland tumor, the MEC, shows MALM-dependent fusions in the vast majority of low-grade cases, while the percentage of gene fusions dramatically drops in high-grade tumors [140]. This observation challenged the habit to classify as subtypes or variants, tumors that are genetically very distinct [141,142,143]. On the other hand, the NGS technic always requires a validation of results (especially for seqRNA approaches) and, therefore, it is likely that a combination of tools (i.e., IHC, FISH, and NGS) has to be used to reach an accurate diagnosis.

### 5.3. Novel Therapeutic Approaches

#### 5.3.1. Molecular Targets

As our knowledge on the molecular mechanisms of salivary gland tumors evolves, novel pathways have been identified and used as targets for the development of novel therapeutic approaches. The MEC tumor has been associated with a high level of EGFR expression, in association with poor prognosis. Inhibition of the EGF pathway sensitizes the tumor mass to ionizing radiations in certain types of head and neck cancers (such as SCC). Once activated, the EGF pathway increases the proliferation rate via the activation of the MAPK-cascade and survival via the PI3K-AKT pathway. Cetuximab is a monoclonal antibody developed to specifically target the EGF receptor and it has been used as complementary therapy in low-grade tumors or as single therapy in metastatic or recurrent cancers of head and neck carcinoma [144,145]. As the EGF pathway is expressed in the basal layer of skin epithelium, hypersensitivity to Cetuximab has been reported with a specific skin toxicity [146]. Instead, head and neck cancer patients did not show increased xerostomia, pain, or mucositis upon Cetuximab administration, and, therefore, treatment can be used in conjunction with other classical therapies, such as radiotherapy [147,148].

For SDCs, several α-HER2 monoclonal antibodies are being tested (Trastuzumab, Pertuzumab) as well as inhibitors of the HER2-tyrosine kinase (e.g., Lapatinib) [149,150,151,152,153].

In AdCC, molecular inhibitors for the frequently altered pathway MYB are currently under development [153]. Multiple kinase inhibitors such as Sorafenib, Lenvatinib, and Axitinib showed a promising response in advanced AdCC (11%, 15%, and 9%, respectively). In particular, the antiangiogenic effect of Levantinib in Phase II clinical trials showed an important antitumoral activity, although relevant side effects that require continuous monitoring have been reported (hypertension, pain, myocardial infarction, and hemorragies) [154,155,156,157].

Further investigations are required to identify unique targets for the development of more efficient drugs.

#### 5.3.2. Immunotherapy

Immunotherapy is a rapidly growing approach that exploits the patient’s own immune system to tackle cancer. Several approaches go under the umbrella of immunotherapy, ranging from the interference with tumor anti-immune strategies, prevention of pro-cancerous conditions, production of genetically engineered immune cells, to the re-shaping of the tumor microenvironment [158].

#### 5.3.3. Checkpoint Inhibitors

Cancers evolve in a self-protective manner, acquiring the capability to control the activity of the immune system. To avoid hyperactivation, the immune system relies on inhibitory checkpoints that dampen the sensitivity of immune cells to a certain stimulus. Cancer cells can activate inhibitory checkpoints, thus limiting anticancer surveillance mechanisms. CTLA4, PD-1, and PD-L1 are central molecules for immune cells inhibition and blocking antibodies have been developed to reactivate the immune function [159,160,161]. For instance, cancer-induced overexpression of PDL1 inhibits T-cell activation, while specific blocking antibodies interfere with this induction and restore T-cell activity to control cancer progression [162,163]. To date, several clinical trials are testing the efficiency of humanized α-PD-1 antibodies in head and neck malignancies (e.g., Pembrolizumab and Nivolumab [164,165] (clinical trials NCT02105636, NCT03132038).

#### 5.3.4. T-Cell Chimeric Antigen Receptor-T (CAR-T)

One of the most promising immunotherapy approaches is the collection of a patient’s own T-cells to modify their antigen receptor and develop a marker-specific immunity [166,167,168]. Engineered T-cells, are amplified in laboratory and transfused back to the patient where they are supposed to continue their amplification step, to ultimately target cancer cells exposing the specific antigen on their surface [169,170]. The main challenge of this therapy is the constitution of a proper receptor, able not only to identify unique targets, but also to stimulate the expansion and survival of CAR-T cells after transfusion. This therapy has shown very positive results in blood disorders, especially for some types of leukemia, and since 2017, CAR-T are an established form of treatment for patients with T-cell acute lymphoblastic leukemia (T-ALL) [171]. Their application on solid tumors remains to be proven and more research is ongoing to understand the real potential of CAR-T in different types of cancers [167,172].

#### 5.3.5. Cancer Vaccine

Traditionally, vaccines are developed by exploiting target macromolecules (i.e., proteins or peptides, nucleic acids, carbohydrates) to induce specific immune reactions. In the context of cancer, the same principle is applied, with cancer cells from patients extracted and used as sources for immunizing antigens. A more advanced approach of cancer vaccine uses antigen-presenting dendritic cells to instruct the immune system on cancer antigens [173,174,175]. Of relevance, this type of immunotherapy is particularly efficient when used as a preventive approach.

Several cancers, including oral and salivary gland cancers, are thought to originate from viral infections. HPV, hepatitis B virus (HBV), and EBV are the most common viral causes leading to cancer development due to their ability to integrate their genomic material and misplace elements of genetic regulation in the host. Vaccines that block HPV and HBV infections in vulnerable organs have been proven efficient in contrasting tumor development in the cervix and liver, hence, are now approved as preventive measure. These vaccines mainly confer a humoral immunity, but recently they have also been associated with stimulating CD4 and cytotoxic T-cell responses [176,177,178].

#### 5.3.6. Tumor Microenvironment

Intracellular alteration alone failed to describe the complexity and the continuous adaptability of cancer. Cancer cells are instead able to continuously interact with their surrounding microenvironment, shaping it to their needs and producing valuable interactions for their own sustainment and growth. Several cell components are part of the tumor microenvironment, including activated fibroblasts, endothelial, and immune cells [179]. The latter are of particular interest because of their regulatory potential [180]. Regulatory T-cells, myeloid-derived suppressor cells (MDSCs), and M2 macrophages promote tumor growth, while M1 macrophages, dendritic cells, NK, and CD8 T-cells function as cancer inhibitors [181,182]. Immunomodulation of the cancer microenvironment, therefore, is a promising approach to disrupt the favorable pro-tumorigenic conditions that allow cancer initiation, establishment, and growth.

## 6. Conclusions

Our understanding of the biology of salivary gland tumors is constantly evolving. Gaining insight on the mechanisms of initiation and progression of these cancers will help in the efforts to develop more efficient diagnostic and treatments tools. Particularly, biomedical studies on novel disease models, screening platforms, and new therapeutic approaches will pave the way for a tailored personalized medicine.

## Supplementary Materials

The following are available online at https://www.mdpi.com/2072-6694/12/11/3107/s1, Table S1: Modified WHO classification list 2017 of SG tumors, including genes and molecular alterations.

## Abbreviations

AciCC	Acinic cell carcinoma
AdCC	Adenoid cystic carcinoma
AP2g	Transcription factor AP-2 gamma
APC	Adenomatous polyposis coli
AQPs	Aquaporins
AREG	Amphiregulin
BCL2	B-cell lymphoma 2
CAR-T	T-cells chimeric antigen receptor-T
CD31	Cluster of differentiation 31
CD4	Cluster of differentiation 4
CD8	Cluster of differentiation 8
CDH5	Cadherin 5/Ve-Cadherin
CMV	Cytomegalovirus
CREB	Coactivator of the cAMP response element-binding protein
CRS	Cytokine release syndrome
CTLA4	Cytotoxic T-lymphocyte-associated protein 4
DLBCL	Diffuse large B-cell lymphoma
EBV	Epstein–Barr virus
ECM	Extra-cellular Matrix
EGF	Epidermal growth factor
ERKs	Extracellular signal-regulated kinases
FGF2	Basic fibroblast growth factor
FISH	Fluorescent in situ hybridization
FLT1	(or VEGFR-1) vascular endothelial growth factor receptor 1
HBV	Hepatitis B virus
Hes1/5	Hairy and enhancer of split 1/5
HIV	Human immunodeficiency viruses (HIV)
HPV	Papillomavirus
HSV	Herpes virus
HTN3-MSANTD3	Histatin3 and Myb/SANT-like DNA-binding domain containing 3
IHC	Immunohistochemistry
IL6	Interleukin 6
JAK/STAT	Janus Kinase/Signal Transducer and Activator of Transcription
Kit	(or SCFR) Stem cell factor receptor
LC3B	Microtubule-associated protein 1 light chain 3 bet
MALM	Mastermind-like protein
MALT	Mucosa-associated lymphoid tissue lymphoma
MAPK	Mitogen-activated protein kinase (MAPK or MAP kinase)
MDSCs	Myeloid-derived suppressor cells
MEC	Mucoepidermoid carcinoma
METC-1	Epidermoid carcinoma translocated-1
MMTV	Mouse mammary tumour virus
MYB	Myeloblastosis
MYBL1	MYB ligand
NFIB	Nuclear Factor I B
NGS	Next-Generation Sequencin
NK	Natural Killers
NR4A2	(or NURR1) The Nuclear receptor related 1 protein
PA	Pleomorphic adenoma
PAC	Polymorphous adenocarcinoma
PD-1	Programmed cell death protein 1
PDGFa/b	Platelet-derived growth factor subunit A
PD-L1	PD1-Ligand
PI3K	Phosphoinositide 3-kinase
PTAH	Phosphotungstic acid–hematoxylin *stain*
PTEN	Phosphatase and tensin homolog
SCC	Squamous cell carcinoma
SOX4	SRY-Box Transcription Factor 4
Wnt	Wingless-related integration site
WWII	World War II
