# Supplementary material for: Molecular and Cellular Modelling of Salivary Gland Tumors Open New Landscapes in Diagnosis and Treatment
Source: Cancers (Basel). 2020 Oct 24;12(11):3107. doi: 10.3390/cancers12113107 (PMC7690880; doi:10.3390/cancers12113107)
Supplement: Supplementary file 1 [file cancers-12-03107-s001.pdf]

cancers

MDPI

Supplementary Materials

Molecular and Cellular Modelling of Salivary Gland Tumors Open New Landscapes in Diagnosis and Treatment

Cristina Porcheri, Christian T. Meisel and Thimios A. Mitsiadis

Table S1. Modified WHO classification list 2017 of SG tumors, including genes and molecular alterations.

Tumor	Type	Gene Rearrangement	Molecular Alterations
**Pleomorphic adenoma**	Benign	t(3;8) (p21;q12)	PLAG1<br>β-catenin
**Myoepithelioma**	Benign		
**Basal Cell adenoma**	Benign		
**Warthin tumour**	Benign		
**Oncocytoma**	Benign		
**Lymphadenoma**	Benign		
**Cystadenoma**	Benign		
**Sialadenoma papilliferum**	Benign		
**Ductal papilloma**	Benign		
**Sebaceous adenoma**	Benign		
**Canalicular adenoma/ductal adenomas**	Benign		
**Sialoblastoma**	Borderline tumor		
**Haemangioma**	Soft tissue lesions		
**Lipoma/sialolipoma**	Soft tissue lesions		
**Nodular fasciitis**	Soft tissue lesions		
**Sclerosing polycystic adenosis**	Epithelial lesions		
**Nodular oncocytic hyperplasia**	Epithelial lesions		
**Lymphoepithelial lesions**	Epithelial lesions		

**Intercalated duct hyperplasia**	Epithelial lesions		
**Acinic cell carcinoma**	Malignant	t(4;9)(q13;q31)<br>HTN3-MSANTD3	NR4A3/Cyclin D1
**Secretory Carcinoma**	Malignant	t(12;15)(p13;q25):<br>ETV6-NTRK3<br>ETV6-RET	
**Mucoepidermoid carcinoma**	Malignant	MALM2/METC1<br>t(11;19)	NOTCH<br>EGF/AREG<br>AQP1/AQP3/AQP5
**Adenoid cystic carcinoma**	Malignant	t(6;9)(q22-23;p23-24)<br>6q23-q27, 12q12-q14<br>1p32-p36	WNT/β-catenin<br>AP2γ/Kit<br>MYB/MYBL1/NFIB<br>P53/p73/CDH5<br>SOX4<br>NOTCH<br>p63/p40
**Polymorphous adenocarcinoma**	Malignant	PRKD1 E710D	Beclin<br>LC3B<br>BCL2<br>Survivin<br>FGF2<br>PDGFα/β
**Epithelial-myoepithelial carcinoma**	Malignant		
**Clear cell carcinoma**	Malignant		
**Basal cell adenocarcinoma**	Malignant		
**Sebaceous adenocarcinoma**	Malignant		
**Intraductal carcinoma**	Malignant		
**Cystadenocarcinoma**	Malignant		
**Adenocarcinoma NOS**	Malignant		
**Salivary duct carcinoma**	Malignant		HER2<br>GCDFP-15<br>p53<br>AR/ERβ

			EGFR<br>TGFα<br>PPARγ
**Myoepithelial carcinoma**	Malignant		
**Carcinoma ex pleomorphic adenoma**	Malignant		
**Carcinosarcoma**	Malignant		
**Poorly differentiated carcinoma**<br>- Neuroendocrine and non-neuroendocrine<br>-Undifferentiated carcinoma<br>-Large cell neuroendocrine carcinoma<br>-Small cell neuroendocrine carcinoma	Malignant		
**Lymphoepithelial carcinoma**	Malignant		
**Squamous cell carcinoma**	Malignant		WNT/β-catenin<br>BMP<br>Notch
**Oncocytic carcinoma**	Malignant		
**Extranodal marginal zone lymphoma of MALT**	Haematolymphoid tumors		

© 2020 by the authors. Licensee MDPI, Basel, Switzerland. This article is an open access article distributed under the terms and conditions of the Creative Commons Attribution (CC BY) license (http://creativecommons.org/licenses/by/4.0/).
